# Prevalence and risk factors for dry eye disease: the Sao Paulo dry eye study

**DOI:** 10.5935/0004-2749.202200100

**Published:** 2025-02-11

**Authors:** Leonardo Guedes C. Marculino, Rossen Mihaylov Hazarbassanov, Nicolle Gilda Teixeira de Queiroz Hazarbassanov, Flavio Hirai, Jose Arthur P. Milhomens Filho, Tais Hitomi Wakamatsu, José A.P. Gomes

**Affiliations:** 1 Department of Ophthalmology and Visual Sciences, Escola Paulista de Medicina, Universidade Federal de São Paulo, São Paulo, SP, Brazil; 2 Pathology Department, Faculdade de Medicina Veterinária e Ciência Animal, Universidade de São Paulo, São Paulo, SP, Brazil

**Keywords:** Dry eye syndromes, ocular surface, Surveys and questionnaires, Síndromes do olho seco, Superfície ocular, Questionários e inquéritos

## Abstract

Purpose: To estimate the prevalence and risk factors of dry eye disease symptoms and clinical diagnoses in Sao Paulo city, state of Sao Paulo, Brazil. Methods: A total of 582 participants over 18 years old, living in the east zone of Sao Paulo city responded to a short questionnaire. Dry eye disease was on that is defined by the presence of severe symptoms or previous clinical diagnosis of dry eye disease by an ophthalmologist. The association between dry eye disease and possible risk factors was assessed. Results: Overall dry eye disease severe symptoms and/ or clinical diagnoses prevalence was calculated as 24.4% for both sexes. Women presented a higher frequency of severe symptoms of dry eye disease (16.07%) than men (8.48%; p=0.0244), as well as the composite of severe symptoms or diagnosed dry eye disease, presented by 26.86% of women and 18.18% of men (p=0.0366). In women, ages between 55 to 75 years old were associated with dry eye disease severe symptoms (OR=3.11; 95%CI 1.56-6.23, p=0.001) and diagnosed dry eye disease (OR=2.02; 95% CI 1.04-3.93, p=0.037). Hypertension was significantly associated with dry eye disease symptoms (OR=1.98; 95% CI 1.14-3.43, p=0.015) and diagnoses (OR=3.54; 95% CI 1.92-6.53, p=0.0001) in women. Eye drops use was associated with severe symptoms of dry eye disease and diagnosed dry eye disease in both women and men (p≤0.01). Conclusions: Dry eye disease prevalence in Sao Paulo city is higher in women than in men. Age and hypertension were stronger risk factors of dry eye disease for women, while eye drops use was a significant indicator of dry eye disease for both sexes.

## Abstract (pt)

**Objetivo:**

Estimar a prevalência e os fatores de risco para os sintomas e o diagnóstico clínico da doença do olho seco na cidade de São Paulo, estado de São Paulo, Brasil.

**Métodos:**

Quinhentos e oitenta e dois participantes acima de 18 anos, residentes na zona leste da cidade de São Paulo responderam a um questionário de três perguntas sobre olho seco. A doença do olho seco foi definida pela presença de sintomas severos ou diagnóstico clínico prévio de doença do olho seco por um oftalmologista. A associação entre doença do olho seco e possíveis fatores de risco foi avaliada.

**Resultados:**

A prevalência de sintomas graves da doença do olho seco e/ ou diagnóstico clínico foi calculada em 24,4% para ambos os sexos. O sexo feminino apresentou uma frequência maior de sintomas severos da doença do olho seco (16,07%) que o sexo masculino (8,48%; p=0,0244), assim como a associação de sintomas severos ou diagnóstico de doença do olho seco, foi de 26,86% no sexo feminino e 18,18% no sexo masculino (p=0,0366). No sexo feminino, a faixa etária entre 55 e 75 anos de idade foi associada com sintomas severos da DOS (odds ratios (OR) = 3,11; IC 95% 1,56-6,23; p=0,001) e com doença do olho seco diagnosticada (OR=2,02; IC 95% 1,043,93; p=0,037). Hipertensão foi associado com sintomas da doença do olho seco (OR=1,98; IC 95% 1,14-3,43; p=0,015) e diagnóstico da doença do olho seco (OR=3,54; IC 95% 1,92-6,53; p=0,001) no sexo feminino. Uso de colírios foi associado a sintomas severos e diagnóstico da doença do olho seco em ambos os sexos (p≤0,01).

**Conclusão:**

A prevalência da doença do olho seco na cidade de São Paulo é mais frequente no sexo feminino que no masculino. Idade e hipertensão foram fatores de risco maiores para doença do olho seco no sexo feminino, enquanto uso de colírios foi um indicador de doença do olho seco para ambos os sexos.

## INTRODUCTION

Dry eye disease (DED) is one the most frequent eye conditions observed in ophthalmological care^(1)^. It has been defined as a complex multifactorial disease accompanied by ocular surface inflammation and high tear osmolarity^(1)^. DED can severely affect an individual’s eye function and quality of life, affecting daily activities, physical and social functions, as well as work productivity^(2,3)^.

Several worldwide population studies have already been performed to evaluate the prevalence of DED^(3-6)^. According to the largest North-American epidemiological DED prevalence estimates, around 7.8% of American women and 4.7% of men aged >50 y has DED^(7,8)^. However, limited data exist on DED prevalence and risk factors in Brazil and other countries of South America^(9)^. Moreover, South American studies have associated dry eye symptoms with conditions, such as vitamin A deficiency xerophthalmia and poor overall nutritional statuses, Hansen’s disease, rheumatoid arthritis, lupus erythematosus, inflammatory bowel disease, HTLV-1 infection, involutional entropion in the elderly, psoriatic arthritis, and primary Sjögren’s syndrome^(10-13)^.

Sao Paulo city is the capital of Sao Paulo state, Brazil, and is considered the southern hemisphere’s most densely populated city^(14)^. Sao Paulo’s municipal area is divided into 5 regions: central, north, south, west, and east. The latest 2007 census reveals that the east zone has the lowest per capita income and highest poverty rate with the lowest human development indexes (values: 0.779, 0.808 and 0.801, respectively)^(15)^. The present study evaluated the prevalence and risk factors for DED in 582 men and women in eastern Sao Paulo city.

## METHODS

### Study subjects

This research was conducted as per the Declaration of Helsinki and was approved by the Ethics Committee of the Paulista School of Medicine - Federal University of Sao Paulo. A total of 3678 eligible participants were selected according to the database used by the Sao Paulo Eye Study (SPES) of Salomao et al.^(16)^ SPES was a population-based study of blindness and associated risk factors prevalence in three low-income neighborhoods in the eastern zone of the city of Sao Paulo: Vila Jacui, Sao Miguel, and Ermelino Matarazzo. The mapping of the geographic area of the study and of all the households within each conglomerate was previously performed by an enumeration and interview team to delimit the perimeter and define the households that could be part of the study^(16)^.

### Questionnaire

A translation and adaptation of the short dry eye questionnaire to Brazilian Portuguese was performed following a multistep approach to ensure its quality^(17)^. The questionnaire was culturally adapted, following the six stage model outlined in previous publications^(18)^, with a few additions and modifications as follows: (1) a literature review on the questionnaire was performed, and an expert committee with translators and ophthalmologists was assembled; (2) the questionnaire was translated from English to Brazilian Portuguese by two translators, one with and one without medical background; (3) a second meeting of the expert committee was held and a synthesis version of the two translations was created; (4) the synthesis version was applied to 30 volunteers and they expressed their comments, difficulties, and improvements for better comprehension; (5) suggestions and necessary modifications were performed to create the pre-final version; (6) a third expert committee verified and approved the final questionnaire version (Table 1); (7) the questionnaire was back-translated to English by two additional translators blinded to the original source text; (8) a final meeting of the expert committee was held and the equivalence of the back-translation with the original text was evaluated.

**Table 1:** Dry eye disease assessment questionnaire

Question 1	(1) “How often do your eyes feel dry? (not wet enough?)”
Question 2	(2) “How often do your eyes feel irritated?”
Possible answers for questions 1 and 2	“constantly” “often”“sometimes”“never”
Question 3	(3) “Have you ever been diagnosed by a clinician as having dry eye syndrome?”
Possible answers for question 3	“yes” “no”

The translated questionnaire was then applied for DED ascertainment, as previously reported^(6-8,19)^. From 3678 eligible subjects, 582 (15.82%) completed the short DED questionnaire. Participants were asked the following 3 questions: (1) “How often do your eyes feel dry? (not wet enough?)”; and (2) “How often do your eyes feel irritated?” Possible answers to these questions included “constantly” (score 3), “often” (score 2), “sometimes” (score 1), or “never” (score 0). Participants who answered “often” or “constantly” for both questions 1 and 2 were considered as having severe DED symptoms. We additionally asked the third question: (3) “Have you ever been diagnosed by a clinician as having dry eye syndrome?” For this question, the participant could answer “Yes” (score 1) or “No” (score 0).

Information on sex, age (stratified as <55 years old, between 55 and 75 years old and >75 years old), educational record (as established by the Brazilian Ministry of Education: incomplete fundamental, complete fundamental, incomplete secondary, complete secondary, incomplete superior, complete superior), ethnicity, computer use (yes/no), cigarette smoking, diagnosis of Diabetes Mellitus type II, history of cardiopathies, hypertension, rheumatism, anti-depressant medication, anti-allergic medication, eye drop, and contact lenses use was gathered.

### Clinical evaluation

All 582 participants who answered the questionnaire were referred for clinical evaluation of DED and were invited to examination. From this group, 51 (35 non-dry eyes and 16 with dry eye severe symptoms and/or dry eye diagnoses) accepted the invitation and were examined. These voluntaries were subjected to the following exams: Ocular Surface Disease Index (OSDI)^(19)^, fluorescein break-up time (FBUT); corneal fluorescein staining; lissamine green staining^(20)^. In addition, ophthalmological exams also included the Schirmer 1 test without anesthesia and tear film osmolarity measured with TearLab^®^ system^(21)^.

### Statistical analysis

The prevalence of DED was calculated, and the corresponding 95% CI was estimated. Using univariate analysis, ORs and 95% CIs of DED for sex, ethnicity, educational degree, lifestyle, and medical factors were recorded. The software used for these analyses was MedCalc version 11, Ostend, Belgium. To compare differences in OSDI, Schirmer I test, FBUT, fluorescein and lissamine staining, and tear film osmolarity between non-DE and DE symptoms and/or DE diagnosed participants, the non-parametric Mann-Whitney test was used. P values of < 0.05 indicated statistically significant differences. These analyses were performed with GraphPad Prism software version 7, San Diego, CA, USA.

## RESULTS

Demographic, lifestyle, and medical history characteristics of participants in the present study are detailed in table 2. The overall prevalence of DED in this population was 24.4% and is shown in table 3. The proportion of women with severe symptoms of DED (16.07%) was higher than that of men (8.48%; p=0.0244), as well as the composite of severe symptoms or diagnosed DED, in which 26.86% of women and 18.18% of men had positive responses for both categories (p=0.0366). However, for clinically diagnosed DED (third question of the short DED questionnaire) there was no sex-based difference in the prevalence (Table 3). Table 4 depicts the analysis of the associations between participant characteristics and severe DED symptoms and diagnoses in women. There was a significant association between age and DED prevalence. The age group between 55 and 75 years old revealed a significant association between severe DED symptoms (OR=3.11; 95% CI 1.56-6.23, p=0.001) and diagnosed DED (OR=2.02; 95% CI 1.043.93, p=0.037), while the age group of >75 years old was associated with diagnosed DED (OR=2.99; 95% CI 1.22-6.85, p=0.016). The analysis also revealed an association between the use of eye drops and hypertension to both symptoms and diagnoses of DED. Among users of ophthalmic solution, there was increased odds of having severe symptoms of DED (OR=2.08; 95% CI 1.193.65, p=0.010) and DED diagnoses (OR=7.74; 95% CI 4.40-13.7, p<0.0001). Hypertension was another characteristic significantly associated with DED symptoms (OR=1.98; 95% CI 1.14-3.43, p=0.015) as well as DED diagnoses (OR=3.54; 95% CI 1.92-6.53, p=0.0001).

**Table 2:** Characteristics of the study population

Variables	Women n (%)	Men n (%)	Total subjects n (%)
Total	417(71.65)	165 (28.35)	582
Age (yrs)			
<55	135 (32.37)	32 (19.39)	167 (28.69)
55-75	231 (55.40)	104 (63.03)	335 (57.56)
>75	51 (12.23)	29 (17.58)	80(13.75)
Education			
Incomplete fundamental	90 (21.58)	42 (25.46)	132 (22.68)
Complete fundamental	130 (31.18)	56 (33.94)	186 (31.96)
Incomplete secondary	29 (6.95)	11 (6.67)	40 (6.87)
Complete secondary	108 (25.90)	43 (26.06)	151 (25.94)
Incomplete superior	12 (2.88)	6 (3.64)	18 (3.09)
Complete superior	48 (11.51)	7 (4.24)	55 (9.45)
Race/ethnicity			
White	256 (61.39)	84 (50.91)	340 (58.42)
African American	28 (6.72)	15 (9.09)	43 (7.39)
Mixed (Mulatto/Pardo)	124 (29.74)	60 (36.36)	184 (31.62)
Asian	5(1.20)	5 (3.03)	10(1.72)
Native American	4 (0.96)	1 (0.61)	5 (0.86)
Computer use	27 (6.48)	8 (4.85)	35 (6.01)
Cigarrette smoking	107 (25.66)	74 (44.85)	181 (31.10)
Diabetes (type 11)	85 (20.38)	31 (18.79)	116 (19.93)
Cardiopathy	35 (8.39)	18 (10.91)	53 (9.11)
Hypertension	223 (53.48)	85 (51.51)	308 (52.92)
Rheumatism	43 (10.31)	8 (4.85)	51 (8.76)
Depression medication	37 (8.87)	9 (5.45)	46 (7.90)
Allergy medication	50(11.99)	9 (5.45)	59 (10.14)
Contact lenses users	6 (1.44)	0 (0.00)	6(1.03)
Eye drop users	98 (23.50)	32 (19.40)	130 (22.34)

**Table 3:** DED symptoms and diagnose prevalence

Severe symptoms	N /total	Prevalence	95% CI
Men	14/165	8.48	5.02-13.84
Women	67/417	16.07	12.84-19.91
Total	81/582	13.92	11.33-16.98
Clinically diagnosed Men	23/165	13.94	9.41-20.11
Women	66/417	15.83	12.62-19.65
Total	89/582	15.29	12.59-18.45
Symptoms or diagnose Men	30/165	18.18	13.00-24.81
Women	112/417	26.86	22.82-31.31
Total	142/582	24.4	21.08-28.05

**Table 4:** Odds ratio association analysis of demographic, lifestyle and medical conditions and DED in women

Variables	Severe symptoms of DED			Clinically siagnosed DED		
	Prevalence (%)	OR (95%C1)	p-value	Prevalence (%)	OR (95%C1)	p-value
Age (yrs)						
<55	11/135 (8.15)	1.00		13/135 (9.63)	1.00	
55-75	50/231 (21.64)	3.11(1.56-6.23)	**0.0013**	41/231 (17.75)	2.02(1.04-3.93)	**0.0373**
>75	6/51 (11.76)	1.5(0.52-4.30)	0.4476	12/51 (23.53)	2.99(1.22-6.85)	**0.0161**
Education						
Incomplete fundamental	12/90 (13.34)	1.00		16/90 (17.78)	1.00	
Complete fundamental	22/130 (16.92)	1.32(0.62-2.83)	0.4699	29/130 (22.31)	1.33(0.67-2.62)	0.4137
Incomplete secondary	8/29 (27.59)	2.48(0.90-6.84)	0.0803	5/29 (17.24)	0.96(0.32-2.9)	0.9475
Complete secondary	18/108 (16.67)	1.3(0.59-2.87)	0.5156	11/108 (10.18)	0.52(0.23-1.2)	0.1253
Incomplete superior	1/12 (8.34)	0.59(0.07-5)	0.6292	0/12 (0)	0.18(0.01-3.2)	0.2436
Complete superior	6/48 (12.5)	0.93(0.32-2.65)	0.8899	5/48 (10.42)	0.54(0.18-1.57)	0.2569
Race/ ethnicity						
White	37/256 (14.45)	1.00		43/256 (16.8)	1.00	
African American	4/28 (14.28)	0.99(0.32-3)	0.9809	6/28 (21.43)	1.35(0.52-3.53)	0.5393
Mixed (Mulatto/ Pardo)	25/124 (20.16)	1.49(0.85-2.62)	0.1597	15/124 (12.1)	0.68(0.36-1.28)	0.2343
Asian	0/5 (0)	0.53(0.03-9.82)	0.6715	1/5 (20)	1.24(0.13-11.35)	0.85
Native American	1/4 (25)	1.97(0.2-19.48)	0.5608	1/4 (25)	1.56(0.17-16.25)	0.6673
Computer use						
No	65/390 (16.67)	1.00		62/390 (20)	1.00	
Yes	2/27 (7.41)	0.4(0.09-1.73)	0.2201	4/27 (14.81)	0.92(0.3-2.75)	0.8815
Cigarrette smoking						
No	49/310 (15.81)	1.00		48/310 (15.48)	1.00	
Yes	18/107 (16.82)	1.08(0.6-1.95)	0.8051	18/107 (16.82)	1.10(0.61-2.0)	0.7437
Diabetes (Type 11)						
No	51/332 (15.36)	1.00		50/332 (15.06)	1.00	
Yes	16/85 (18.82)	1.28(0.69-2.37)	0.4388	16/85 (18.82)	1.31(0.70-2.43)	0.3973
Cardiopathy						
No	58/382 (15.18)	1.00		57/382 (14.92)	1.00	
Yes	9/35 (25.71)	1.93(.86-4.34)	0.1096	9/35 (25.71)	1.97(0.88-4.43)	0.0993
Hypertension						
No	22/194 (11.34)	1.00		15/194 (7.73)	1.00	
Yes	45/223 (19.73)	1.98(1.14-3.43)	**0.0154**	51/223 (22.87)	3.54(1.92-6.53)	**0.0001**
Rheumatism						
No	60/374 (16.04)	1.00		57/374 (15.24)	1.00	
Yes	7/43 (16.28)	1.02(0.43-2.39)	0.9681	9/43 (20.93)	1.47(0.67-3.23)	0.3355
Depression medication						
No	63/380 (16.58)	1.00		63/380 (16.58)	1.00	
Yes	4/37 (10.81)	0.71(0.21-1.78)	0.3661	3/37(8.11)	0.44(0.13-1.49)	0.1888
Allergy medication						
No	62/367 (16.89)	1.00		61/367 (16.62)	1.00	
Yes	5/50(10)	0.55(0.21-1.43)	0.2191	5/50(10)	0.56(0.21-1.46)	0.2346
Contact lenses users						
No	65/411 (15.81)	1.00		65/411 (15.81)	1.00	
Yes	2/6 (33.34)	2.66(0.48-14.83)	0.2641	1/6 (16.67)	1.06(0.12-9.26)	0.9548
Eye drop users						
No	43/319 (13.48)	1.00		26/319 (7.52)	1.00	
Yes	24/98 (24.49)	2.08(1.19-3.65)	**0.0105**	40/98 (40.82)	7.74(4.40-13.72)	**<0.0001**

In men, only the use of eye drops was significantly associated with clinically diagnosed DED or severe symptoms of DED (Table 5). The univariate analysis revealed that eye drop usage is associated with severe symptoms of DED (OR=10.02; 95% CI 3.08-32.60, p=0.0001) and diagnosed DED (OR=18.00; 95% CI 6.43-50.38, p<0.0001).

**Table 5:** Odds ratio association analysis of demographic, lifestyle and medical conditions and DED in men

Variables	Severe symptoms of DED			Clinically diagnosed DED		
	Prevalence (%)	OR (95%C1)	p-value	Prevalence (%)	OR (95%C1)	p-value
Age (yrs)						
<55	3/32 (9.38)	1.00		6/32 (18.75)	1.00	
55-75	9/104 (8.65)	0.93(0.23-3.68)	0.9241	11/104 (10.58)	0.51(0.17-1.52)	0.2276
>75	2/29 (6.90)	0.71(0.11-4.62)	0.7255	6/29 (20.69)	1.13(0.32-4.00)	0.8491
Education						
Incomplete fundamental	4/42 (9.52)	1.00		7/42 (16.67)	1.00	
Complete fundamental	7/56(12.50)	1.35(0.37-4.97)	0.6451	9/56 (6.07)	0.96(0.32-2.82)	0.9371
Incomplete secondary	0/11 (0.00)	0.37(0.02-7.43)	0.5175	0/11 (0.00)	0.20(0.01-3.89)	0.2918
Complete secondary	3/43 (6.98)	0.71(0.14-3.39	0.6705	6/43 (13.95)	0.81(0.25-2.65)	0.7285
Incomplete superior	0/6 (0.00)	0.66(0.03-13.73)	0.7872	0/6 (0.00)	0.36(0.02-7.18)	0.5067
Complete superior	0/7 (0.00)	0.57(0.03-11.74)	0.716	1/7 (14.29)	0.83(0.09-8.04)	0.8748
Race/ethnicity						
White	7/84 (8.33)	1.00		10/84 (11.90)	1.00	
African American	0/15 (0.00)	0.33(0.02-6.14)	0.46	2/15 (13.33)	1.14(0.22-5.80)	0.8706
Mixed (Mulatto/ Pardo)	6/60 (10.00)	1.22(0.38-3.83)	0.7311	10/60 (16.67)	1.48(0.57-3.81)	0.4172
Asian	1/5 (20.00)	2.75(0.26-28.09)	0.3936	1/5 (20)	1.85(0.19-18.24)	0.5983
Native American	0/1 (0.00)	3.44(0.13-92.21)	0.4609	0/1 (0.00)	2.36(0.09-61.93)	0.6054
Computer use						
No	14/157 (8.92)	1.00		21/157 (13.38)	1.00	
Yes	0/8 (0.00)	0.58(0.03-10.61)	0.7149	2/8 (25.00)	2.16(0.41-11.41)	0.365
Cigarrette smoking						
No	11/91 (12.09)	1.00		13/91 (14.29)	1.00	
Yes	3/74 (4.05)	0.30(0.08-1.14)	0.0789	10/74 (13.51)	0.94(0.38-2.28)	0.8868
Diabetes (Type 11)						
No	12/134 (8.96)	1.00		21/134 (15.67)	1.00	
Yes	2/31 (6.45)	0.70(0.14-3.30)	0.6536	2/31 (6.45)	0.37(0.08-1.67)	0.1972
Cardiopathy						
No	12/147 (8.16)	1.00		19/147 (12.93)	1.00	
Yes	2/18 (11.11)	1.5(0.31-7.46)	0.5979	4/18 (22.22)	1.92(0.57-6.46)	0.2893
Hypertension						
No	8/80 (10.00)	1.00		8/80 (10.00)	1.00	
Yes	6/85 (7.06)	0.68(0.23-2.06)	0.5	15/85 (17.65)	1.92(0.77-4.83)	0.1613
Rheumatism						
No	12/157 (7.64)	1.00		23/157 (15.65)	1.00	
Yes	2/8 (25.00)	4.03(0.73-22.16)	0.1093	0/8 (0.00)	0.33(0.02-6.03)	0.4596
Depression medication						
No	14/156 (8.97)	1.00		21/156 (13.46)	1.00	
Yes	0/9 (0.00)	0.57(0.03-10.26)	0.702	2/9 (22.22)	1.83(0.36-9.44)	0.4667
Allergy medication						
No	13/156 (8.33)	1.00		22/156 (14.10)	1.00	
Yes	1/9 (11.11)	1.5(0.17-12.94)	0.7122	1/9 (11.11)	0.76(0.09-6.39)	0.8017
Contact lenses users						
No	14/165 (8.48)	1.00		23/165 (13.94)	1.00	
Yes	0/0 (0.00)	10.45(0.2-546.37)	0.2451	0/0 (0.00)	6.06(0.12-313.11)	0.3704
Eye drop users						
No	5/133 (3.76)	1.00		7/133 (5.26)	1.00	
Yes	9/32 (28.13)	10.02(3.08-32.60)	**0.0001**	16/32 (50.00)	18.00(6.43-50.38)	**<0.0001**

For both sexes, no significant association between DED and ethnic group, level of education, and other characteristics of study participants was observed.

**Figure 1:**
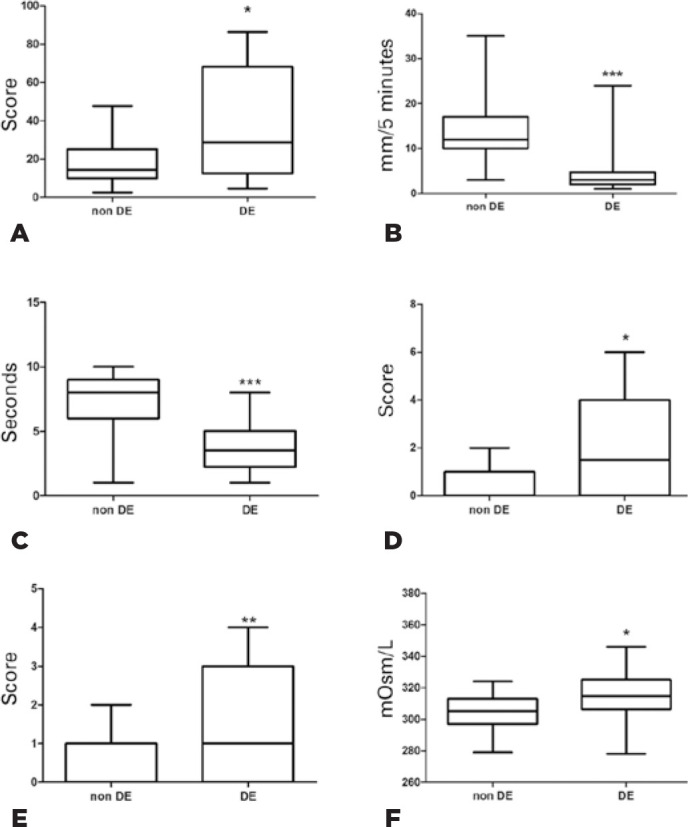
Ophthalmological exams. Groups were divided according to the dry eye short questionnaire responses. Non-DE are participants that did not present severe symptoms nor a previous DED diagnosis by a clinician are included above. DE participants had severe symptoms of DED and/ or were previously diagnosed of DED. (A) Ocular surface disease index (OSDI) scores were significantly higher in DED participants than non-DED participants. *Mann-Whitney, p=0.0275. (B) Schirmer I values were significantly lower for DE participants. ***Mann-Whitney, p=0.0002. (C) FBUT values were significantly lower for DE participants. ***Mann-Whitney, p < 0.0001. (D) Corneal fluorescein staining values were significantly higher for DE participants. *Mann-Whitney, p=0.0405. (E) Lissamine staining values were significantly higher for DE participants. **Mann-Whitney, p=0.0022. (F) Tear film osmolarity values were significantly higher for DE participants. *Mann-Whitney, p =0.0164.

Following phone interviews, participants were invited for ophthalmological exams. The analysis of ophthalmological tests results was divided according to the following groups: (1) non-DED, (2) composite of DED severe symptoms and/or previous DED diagnoses. OSDI, fluorescein and lissamine corneal staining and tear osmolarity values were significantly higher for DED severe symptoms and/or DED diagnosed participant group when compared to non-DE participants (Mann-Whitney test, p<0.05) (Figure 1A, D, E and F) Whereas Schirmer I and FBUT measurements were lower for DED severe symptoms and/or DED diagnosed participants, and were statistically significant when compared to non-DED participants (Mann-Whitney test, p<0.05) (Figure 1B and C).

## DISCUSSION

According to the Epidemiology subcommittee of the first Dry Eye Workshop (DEWS), the prevalence of DED was estimated to range from 5 to 30% in individuals over 50 years old^(1)^. In two main North-American studies, the Women’s Health Study (WHS) and the Physician’s Health Study (PHS), Schaumberg and collaborators used the short dry eye questionnaire to evaluate the prevalence of DED in women and men. In the WHS, the prevalence of DED severe symptoms in women was estimated to be 3.4%, the prevalence of clinically diagnosed DED as 4.7%, and the composite of severe symptoms or clinically diagnosed DED as 6.7%^(7)^. The same questionnaire and criteria were applied to the PHS, which found that the prevalence of DED severe symptoms in men was 2.2%, clinically diagnosed DED was 3.0%, and the composite of symptoms and diagnosed DED was 4.3%^(8)^. In contrast to the North-American results, studies from Asia revealed that the prevalence of symptoms or diagnosed DED in adults was estimated to be 12.5% in men and 21.6% in women^(6)^, while other studies calculated the overall prevalence to range from 27.5 to 34%^(4,5)^. In the Brazilian cohort study, the previous diagnosis was reported by 10.2% and presence of severe symptoms in 4.9% of DED patients^(22)^. Our study showed an overall prevalence of 24% for severe DED symptoms and/or clinical diagnoses for both the sexes.

Castro et al.^(22)^ reported the first study about prevalence and risk factors of dry eye in a large population sample from all regions of Brazil. They found that prevalence of DED is common, and its rates vary substantially in the different geographic regions of the country. The present study evaluates DED symptoms and clinical signs in a large city that was not previously reported.

Sao Paulo city is the largest in South America, with a population of more than 12 million people and significant ethnic diversity, as well as educational and economic disparities. Sao Paulo has a subtropical climate and polluted air during most of the year, particularly in the dry season from April to early September.

Our population-based study found that the overall prevalence of DED is 24.4%, while severe DED symptoms in women and men is 16.07% and 8.48%, respectively. The prevalence of clinically diagnosed DED is 15.83% in women and 13.84% in men, while the composite of symptoms and clinical diagnoses of DED is 26.86% for women and 18.18% for men.

Castro et al. assessed some main factors associated with DED, such as sex, age, ocular surgery history, contact lens use, cancer treatment, computer use, antidepressants, and anti-allergy medications^(22)^. In our study, we estimated the prevalence of DED and risk factors for patients aged > 55 y, hypertension in women, and eye drops use both sexes.

The prevalence of DED calculated by the present study is consistent with the rates reported in DEWS^(1)^. However, the calculated values are lower than Asian studies^(4-6)^ and considerably higher than the numbers found in WHS, PHS and Castro et al. studies^(7,8,22)^. This discrepancy may be explained by the location of our study population, which consists of a highly industrialized area with increased pollution levels^(23-25)^, as well as low relative humidity^(26,27)^.

Odds ratio analyses in this study showed an age-related trend for DED symptoms and diagnoses in women between 55-75 years old, but not for men. This finding has also been reported by Uchino et al.^(6)^ and the WHS^(7)^. Additionally, as described by Uchino and collaborators, the absence of a trend for DED symptoms in female participants over 75 years old may be related to an underestimation of symptoms due to concomitant systemic disorders or a decrease in cornea sensitivity.

The DEWS report has detailed that the use of some medications may be considered risk factors for DED, such as antihistamines, beta-blockers, antispasmodics, and diuretics^(1,28)^. Our finding of an increased ratio of DED symptoms and diagnoses in female participants that also reported having hypertension may partially be explained by the use of medications such as beta-blockers or diuretics. Other studies have found a relationship between hypertension and DED in men, but not women^(6,8)^. Our study estimated a trend (p<0.2) for DED diagnoses in hypertensive men; however it is likely that our low participation rate among men in this population hindered the observation of a significant association between hypertension and DED.

In the present study, both DED symptoms and clinically diagnosed DED were significantly associated with the use of eye drops by women and men. Beyond the probable use of artificial tears and lubricants, it is possible that these participants also used other categories of eye drops such as vasoconstrictors and glaucoma medications. The use of such types of eye drops was associated with a higher incidence of ocular symptoms and signs than preservative-free drops^(29)^. Our study was not designed to address the differences in eye drop use.

In regard to ophthalmological exams data, significant differences in OSDI, tear film osmolarity, Schirmer I, FBUT, fluorescein and lissamine staining values were found between non-DED participants, and the composite group of participants with DED severe symptoms and/or clinically diagnosed DED. These findings further confirm the validity of the short dry eye questionnaire. In the Castro et al. study^(30)^ was restricted only to patient self-report of symptoms or previous diagnosis of the disease using a short questionnaire.

No association between DED symptoms and diagnoses, and race or educational level was found. There was also no association between factors previously described in other articles, including cigarette smoking, computer use, contact lens use, myocardial infarction or angina (cardiopathy), depression, and allergy medication^(1,6)^. Although some of these risk factors are not relevant for this specific population, such as computer use and contact lens use, it is likely that the other risk factors may require a larger sample in order to adequately ascertain their association to DED symptoms and diagnoses within a Sao Paulo - Brazilian cohort.

Some potential limitations of this study must be pointed out. This work cannot be considered a national population-based study once the city of São Paulo is not representative of the Brazilian population, but we believe that it brings consistent information once it includes patients in which dry eye was objectively diagnosed by clinical evaluation and reliable diagnosis tests.

In conclusion, in this epidemiological study we aimed to evaluate DED symptoms and clinically diagnosed DED in Brazil’s largest city, in which a DED prevalence of approximately 24% was recorded. Ages over 55 years old and hypertension appear to be significant risk factors for DED in Sao Paulo female residents.

## References

[1] (2007). The definition and classification of dry eye disease: report of
the Definition and Classification Subcommittee of the International Dry Eye
WorkShop (2007). Ocul Surf.

[2] Pflugfelder SC. (2008). Prevalence, burden, and pharmacoeconomics of dry eye
disease. Am J Manag Care.

[3] Paulsen AJ, Cruickshanks KJ, Fischer ME, Huang GH, Klein BE, Klein R (2014). Dry eye in the beaver dam offspring study: prevalence, risk
factors, and health-related quality of life. Am J Ophthalmol.

[4] Lee AJ, Lee J, Saw SM, Gazzard G, Koh D, Widjaja D (2002). Prevalence and risk factors associated with dry eye symptoms: a
population based study in Indonesia. Br J Ophthalmol.

[5] Lin PY, Tsai SY, Cheng CY, Liu JH, Chou P, Hsu WM. (2003). Prevalence of dry eye among an elderly Chinese population in
Taiwan: the Shihpai Eye Study. Ophthalmology.

[6] Uchino M, Nishiwaki Y, Michikawa T, Shirakawa K, Kuwahara E, Yamada M (2011). Prevalence and risk factors of dry eye disease in Japan: koumi
study. Ophthalmology.

[7] Schaumberg DA, Sullivan DA, Buring JE, Dana MR. (2003). Prevalence of dry eye syndrome among US women. Am J Ophthalmol.

[8] Schaumberg DA, Dana R, Buring JE, Sullivan DA. (2009). Prevalence of dry eye disease among US men: estimates from the
Physicians’ Health Studies. Arch Ophthalmol.

[9] Rios JL, Boechat JL, Gioda A, dos Santos CY, de Aquino Neto FR, Lapa e Silva JR (2009). Symptoms prevalence among office workers of a sealed versus a
non-sealed building: associations to indoor air quality. Environ Int.

[10] Roncada MJ, Wilson D, Mazzilli RN, Gandra YR. (1981). [Hypovitaminosis A in communities of the State of São
Paulo, Brazil]. Rev Saude Publica.

[11] Castro-Lima Vargens C, Grassi MF, Boa-Sorte N, Rathsam-Pinheiro RH, Olavarria VN, de Almeida Kruschewsky R (2011). Keratoconjunctivitis sicca of human T cell lymphotropic virus
type 1 (HTLV-1) infected individuals is associated with high levels of
HTLV-1 proviral load. J Clin Virol.

[12] Lima FB, Abalem MF, Ruiz DG, Gomes BA, Azevedo MN, Moraes Jr, HV (2012). Prevalence of eye disease in Brazilian patients with psoriatic
arthritis. Clinics (São Paulo).

[13] Valim V, Zandonade E, Pereira AM, de Brito Filho OH, Serrano EV, Musso C (2013). Primary Sjögren’s syndrome prevalence in a major
metropolitan area in Brazil. Rev Bras Reumatol.

[14] Instituto Brasileiro de Geografia e Estatística
(IBGE) (2010). 2010 Census.

[15] São Paulo (cidade) (2007). Prefeitura Municipal. Atlas Municipal.

[16] Salomao SR, Cinoto RW, Berezovsky A, Araujo-Filho A, Mitsuhiro MR, Mendieta L (2008). Prevalence and causes of vision impairment and blindness in older
adults in Brazil: the Sao Paulo Eye Study. Ophthalmic Epidemiol.

[17] Acquadro C, Conway K, Hareendran A, Aaronson N, European Regulatory Issues and Quality of Life Assessment (ERIQA)
Group (2008). Literature review of methods to translate health-related quality
of life questionnaires for use in multinational clinical
trials. Value Health.

[18] Beaton DE, Bombardier C, Guillemin F, Ferraz MB. (2000). Guidelines for the process of cross-cultural adaptation of
self-report measures. Spine.

[19] Schiffman RM, Christianson MD, Jacobsen G, Hirsch JD, Reis BL. (2000). Reliability and validity of the Ocular Surface Disease
Index. Arch Ophthalmol.

[20] Sullivan BD, Whitmer D, Nichols KK, Tomlinson A, Foulks GN, Geerling G (2010). An objective approach to dry eye disease severity. Invest Ophthalmol Vis Sci.

[21] Lemp MA, Crews LA, Bron AJ, Foulks GN, Sullivan BD. (2012). Distribution of aqueous-deficient and evaporative dry eye in a
clinic-based patient cohort: a retrospective study. Cornea.

[22] Castro JS, Selegatto IB, Castro RS, Miranda EC, de Vasconcelos JP, de Carvalho KM (2018). Prevalence and Risk Factors of self-reported dry eye in Brazil
using a short symptom questionnaire. Sci Rep.

[23] Versura P, Profazio V, Cellini M, Torreggiani A, Caramazza R. (1999). Eye discomfort and air pollution. Ophthalmologica.

[24] Bourcier T, Viboud C, Cohen JC, Thomas F, Bury T, Cadiot L (2003). Effects of air pollution and climatic conditions on the frequency
of ophthalmological emergency examinations. Br J Ophthalmol.

[25] Torricelli AA, Novaes P, Matsuda M, Braga A, Saldiva PH, Alves MR (2013). Correlation between signs and symptoms of ocular surface
dysfunction and tear osmolarity with ambient levels of air pollution in a
large metropolitan area. Cornea.

[26] Paschides CA, Stefaniotou M, Papageorgiou J, Skourtis P, Psilas K. (1998). Ocular surface and environmental changes. Acta Ophthalmol Scand.

[27] Tesón M, López-Miguel A, Neves H, Calonge M, González-García MJ, González-Méijome JM. (2015). Influence of Climate on Clinical Diagnostic Dry Eye Tests: pilot
Study. Optom Vis Sci.

[28] Moss SE, Klein R, Klein BE. (2004). Incidence of dry eye in an older population. Arch Ophthalmol.

[29] Jaenen N, Baudouin C, Pouliquen P, Manni G, Figueiredo A, Zeyen T. (2007). Ocular symptoms and signs with preserved and preservative-free
glaucoma medications. Eur J Ophthalmol.

[30] Castro JS, Selegatto IB, Castro RS, Vasconcelos JP, Arieta CE, Alves M. (2017). Translation and validation of the Portuguese version of a dry eye
disease symptom questionnaire. Arq Bras Oftalmol.

